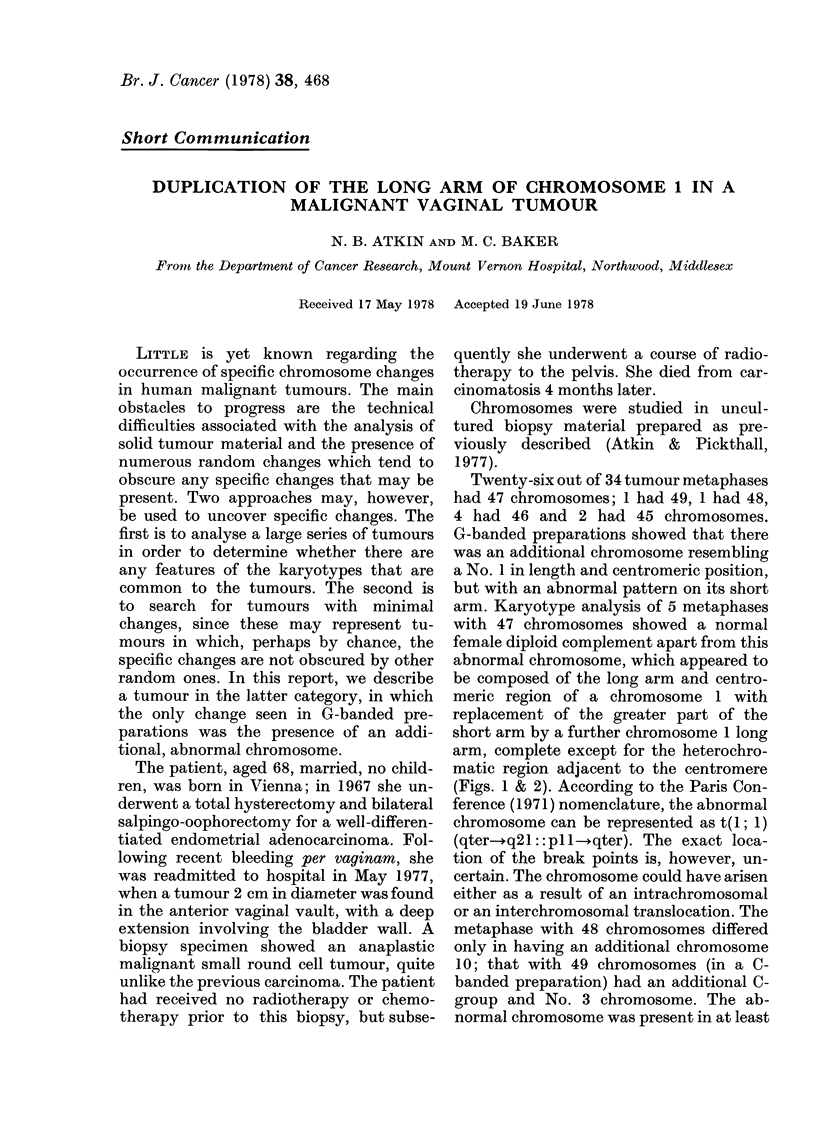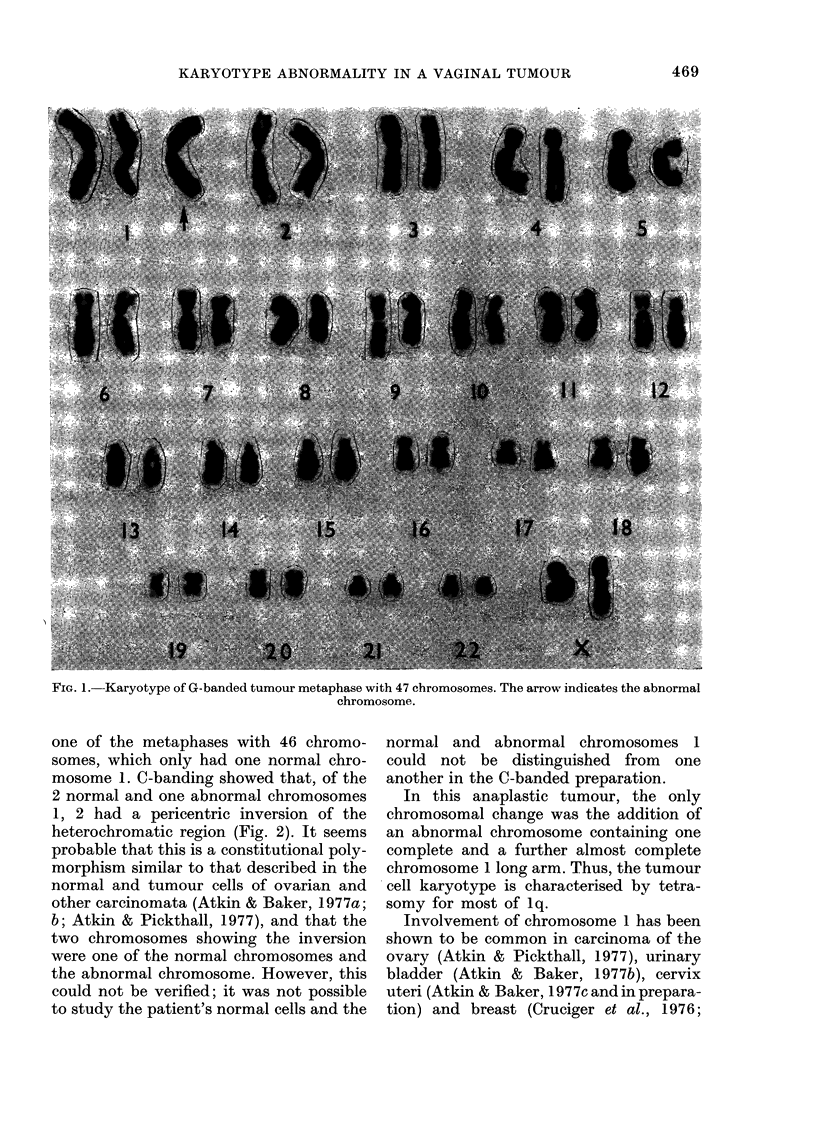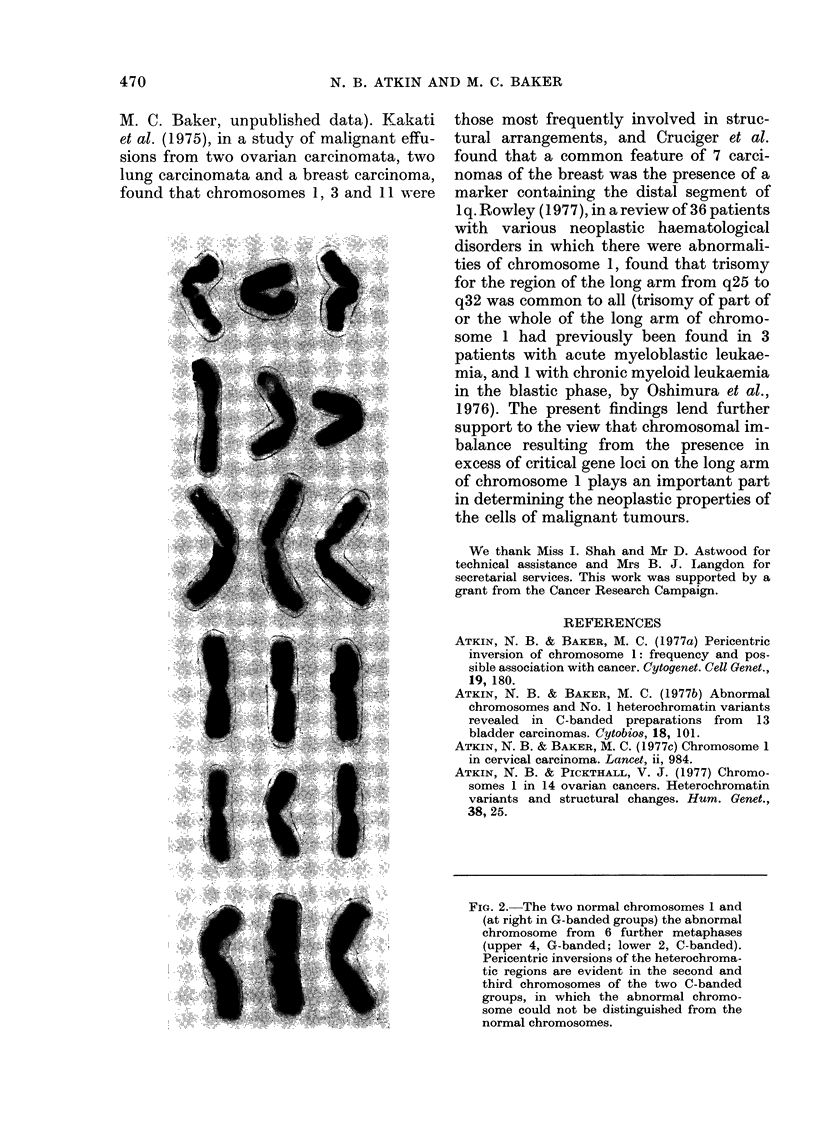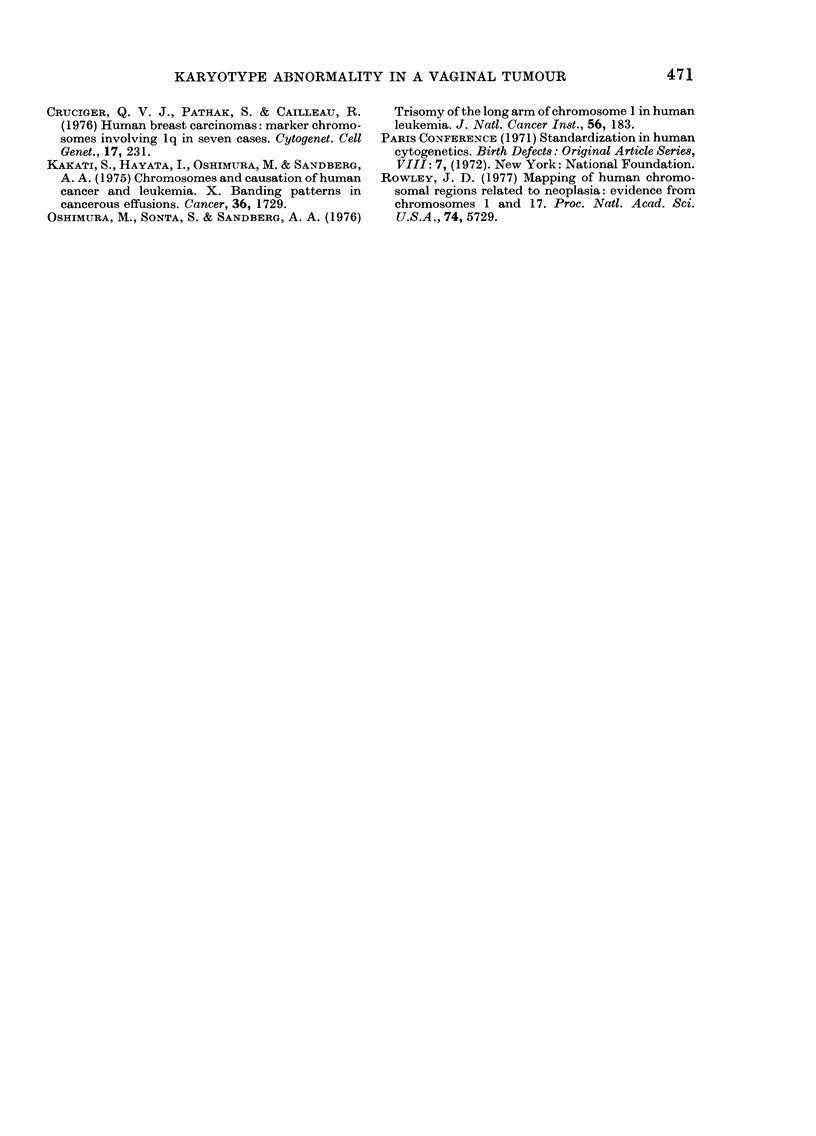# Duplication of the long arm of chromosome 1 in a malignant vaginal tumour.

**DOI:** 10.1038/bjc.1978.232

**Published:** 1978-09

**Authors:** N. B. Atkin, M. C. Baker

## Abstract

**Images:**


*Br. J. Cancer* (1978) **38**, 468

***Short Communication***

### DUPLICATION OF THE LONG ARM OF CHROMOSOME 1 IN A MALIGNANT VAGINAL TUMOUR

N. B. ATKIN AND M. C. BAKER

*From the Department of Cancer Research, Mount Vernon Hospital, Northwood, Middlesex*

Received 17 May 1978 Accepted 19 June 1978

LITTLE is yet known regarding the occurrence of specific chromosome changes in human malignant tumours. The main obstacles to progress are the technical difficulties associated with the analysis of solid tumour material and the presence of numerous random changes which tend to obscure any specific changes that may be present. Two approaches may, however, be used to uncover specific changes. The first is to analyse a large series of tumours in order to determine whether there are any features of the karyotypes that are common to the tumours. The second is to search for tumours with minimal changes, since these may represent tumours in which, perhaps by chance, the specific changes are not obscured by other random ones. In this report, we describe a tumour in the latter category, in which the only change seen in G-banded preparations was the presence of an additional, abnormal chromosome.

The patient, aged 68, married, no children, was born in Vienna; in 1967 she underwent a total hysterectomy and bilateral salpingo-oophorectomy for a well-differentiated endometrial adenocarcinoma. Following recent bleeding *per vaginam*, she was readmitted to hospital in May 1977, when a tumour 2 cm in diameter was found in the anterior vaginal vault, with a deep extension involving the bladder wall. A biopsy specimen showed an anaplastic malignant small round cell tumour, quite unlike the previous carcinoma. The patient had received no radiotherapy or chemotherapy prior to this biopsy, but subsequently she underwent a course of radiotherapy to the pelvis. She died from carcinomatosis 4 months later.

Chromosomes were studied in uncultured biopsy material prepared as previously described (Atkin & Pickthall, 1977).

Twenty-six out of 34 tumour metaphases had 47 chromosomes; 1 had 49, 1 had 48, 4 had 46 and 2 had 45 chromosomes. G-banded preparations showed that there was an additional chromosome resembling a No. 1 in length and centromeric position, but with an abnormal pattern on its short arm. Karyotype analysis of 5 metaphases with 47 chromosomes showed a normal female diploid complement apart from this abnormal chromosome, which appeared to be composed of the long arm and centromeric region of a chromosome 1 with replacement of the greater part of the short arm by a further chromosome 1 long arm, complete except for the heterochromatic region adjacent to the centromere (Figs. 1 & 2). According to the Paris Conference (1971) nomenclature, the abnormal chromosome can be represented as t(1; 1) (qter→q21::p11→qter). The exact location of the break points is, however, uncertain. The chromosome could have arisen either as a result of an intrachromosomal or an interchromosomal translocation. The metaphase with 48 chromosomes differed only in having an additional chromosome 10; that with 49 chromosomes (in a C-banded preparation) had an additional C-group and No. 3 chromosome. The abnormal chromosome was present in at least

FIG. 1.—Karyotype of G-banded tumour metaphase with 47 chromosomes. The arrow indicates the abnormal chromosome.

one of the metaphases with 46 chromosomes, which only had one normal chromosome 1. C-banding showed that, of the 2 normal and one abnormal chromosomes 1, 2 had a pericentric inversion of the heterochromatic region (Fig. 2). It seems probable that this is a constitutional polymorphism similar to that described in the normal and tumour cells of ovarian and other carcinomata (Atkin & Baker, 1977*a*; *b*; Atkin & Pickthall, 1977), and that the two chromosomes showing the inversion were one of the normal chromosomes and the abnormal chromosome. However, this could not be verified; it was not possible to study the patient's normal cells and the normal and abnormal chromosomes 1 could not be distinguished from one another in the C-banded preparation.

In this anaplastic tumour, the only chromosomal change was the addition of an abnormal chromosome containing one complete and a further almost complete chromosome 1 long arm. Thus, the tumour cell karyotype is characterised by tetrasomy for most of 1q.

Involvement of chromosome 1 has been shown to be common in carcinoma of the ovary (Atkin & Pickthall, 1977), urinary bladder (Atkin & Baker, 1977*b*), cervix uteri (Atkin & Baker, 1977*c* and in preparation) and breast (Cruciger *et al.*, 1976;

M. C. Baker, unpublished data). Kakati *et al.* (1975), in a study of malignant effusions from two ovarian carcinomata, two lung carcinomata and a breast carcinoma, found that chromosomes 1, 3 and 11 were those most frequently involved in structural arrangements, and Cruciger *et al.* found that a common feature of 7 carcinomas of the breast was the presence of a marker containing the distal segment of 1q. Rowley (1977), in a review of 36 patients with various neoplastic haematological disorders in which there were abnormalities of chromosome 1, found that trisomy for the region of the long arm from q25 to q32 was common to all (trisomy of part of or the whole of the long arm of chromosome 1 had previously been found in 3 patients with acute myeloblastic leukaemia, and 1 with chronic myeloid leukaemia in the blastic phase, by Oshimura *et al.*, 1976). The present findings lend further support to the view that chromosomal imbalance resulting from the presence in excess of critical gene loci on the long arm of chromosome 1 plays an important part in determining the neoplastic properties of the cells of malignant tumours.

We thank Miss I. Shah and Mr D. Astwood for technical assistance and Mrs B. J. Langdon for secretarial services. This work was supported by a grant from the Cancer Research Campaign.

### REFERENCES

ATKIN, N. B. & BAKER, M. C. (1977*a*) Pericentric inversion of chromosome 1: frequency and possible association with cancer. *Cytogenet. Cell Genet.*, **19**, 180.

ATKIN, N. B. & BAKER, M. C. (1977*b*) Abnormal chromosomes and No. 1 heterochromatin variants revealed in C-banded preparations from 13 bladder carcinomas. *Cytobios*, **18**, 101.

ATKIN, N. B. & BAKER, M. C. (1977*c*) Chromosome 1 in cervical carcinoma. *Lancet*, ii, 984.

ATKIN, N. B. & PICKTHALL, V. J. (1977) Chromosomes 1 in 14 ovarian cancers. Heterochromatin variants and structural changes. *Hum. Genet.*, **38**, 25.

FIG. 2.—The two normal chromosomes 1 and (at right in G-banded groups) the abnormal chromosome from 6 further metaphases (upper 4, G-banded; lower 2, C-banded). Pericentric inversions of the heterochromatic regions are evident in the second and third chromosomes of the two C-banded groups, in which the abnormal chromosome could not be distinguished from the normal chromosomes.

CRUCIGER, Q. V. J., PATHAK, S. & CAILLEAU, R. (1976) Human breast carcinomas: marker chromosomes involving 1q in seven cases. *Cytogenet. Cell Genet.*, **17,** 231.

KAKATI, S., HAYATA, I., OSHIMURA, M. & SANDBERG, A. A. (1975) Chromosomes and causation of human cancer and leukemia. X. Banding patterns in cancerous effusions. *Cancer*, **36,** 1729.

OSHIMURA, M., SONTA, S. & SANDBERG, A. A. (1976) Trisomy of the long arm of chromosome 1 in human leukemia. *J. Natl. Cancer Inst.*, **56,** 183.

PARIS CONFERENCE (1971) Standardization in human cytogenetics. *Birth Defects: Original Article Series, VIII*: **7,** (1972). New York: National Foundation.

ROWLEY, J. D. (1977) Mapping of human chromosomal regions related to neoplasia: evidence from chromosomes 1 and 17. *Proc. Natl. Acad. Sci. U.S.A.*, **74,** 5729.